# Inhibition of GSK‐3*β* increases trabecular bone volume but not cortical bone volume in adenine‐induced uremic mice with severe hyperparathyroidism

**DOI:** 10.14814/phy2.13010

**Published:** 2016-11-02

**Authors:** Narihito Tatsumoto, Masaki Arioka, Shunsuke Yamada, Fumi Takahashi‐Yanaga, Masanori Tokumoto, Kazuhiko Tsuruya, Takanari Kitazono, Toshiyuki Sasaguri

**Affiliations:** ^1^ Department of Clinical PharmacologyFaculty of Medical SciencesKyushu UniversityFukuokaJapan; ^2^ Department of Medicine and Clinical ScienceGraduate School of Medical SciencesKyushu UniversityFukuokaJapan; ^3^ Global Medical Science Education UnitFaculty of Medical SciencesKyushu UniversityFukuokaJapan; ^4^ Department of Internal MedicineFukuoka Dental CollegeFukuokaJapan; ^5^ Department of Integrated Therapy for Chronic Kidney DiseaseGraduate School of Medical SciencesKyushu UniversityFukuokaJapan

**Keywords:** Bone fracture, chronic kidney disease, glycogen synthase kinase‐3*β*, Wnt/*β*‐catenin signaling pathway

## Abstract

Patients with chronic kidney disease (CKD) are at increased risk for bone fractures compared with the general population. Repression of the Wnt/*β*‐catenin signaling pathway is associated with bone abnormalities. Inhibition of glycogen synthase kinase (GSK)‐3*β*, a critical component of the Wnt/*β*‐catenin signaling pathway, increases bone volume through accumulation of *β*‐catenin. It remains unknown whether inhibition of GSK‐3*β* increases bone volume in CKD. The present in vivo study examined the effects of GSK‐3*β* inhibition on bone volume in CKD mice. Wild‐type mice were divided into three groups. One group was fed a control diet (CNT) and the other two groups were fed a diet containing 0.2% adenine and given water with or without lithium chloride (LiCl), a GSK‐3 inhibitor (CKD, CKD+LiCl, respectively). GSK‐3*β* heterozygous knockout mice were fed a diet containing 0.2% adenine (CKD‐GSK‐3*β* ^+/−^). After 6 weeks, trabecular and cortical bone volumes of the femur were analyzed using microcomputed tomography. CKD mice developed azotemia, hyperphosphatemia, and hyperparathyroidism, followed by a decrease in cortical bone volume without any change in trabecular bone volume. Serum levels of urea nitrogen, phosphate, and parathyroid hormone were comparable among the three groups of CKD mice. Trabecular bone volume increased in CKD‐GSK‐3*β* ^+/−^ and CKD+LiCl mice compared with CNT and CKD mice. However, there were no significant differences in cortical bone volume among the three groups of CKD mice. The results suggest that inhibition of GSK‐3*β* increases trabecular bone volume but not cortical bone volume in adenine‐induced uremic mice with uncontrolled hyperparathyroidism.

## Introduction

The incidence of bone fractures is high in patients with chronic kidney disease (CKD) (Alem et al. 2000; Nickolas et al. 2006; Ensrud et al. 2007; Fried et al. 2007), and this increased incidence is associated with high rates of hospitalization, disability, and mortality in patients with CKD (Mittalhenkle et al. 2004; Nitsch et al. 2009; Tentori et al. 2014). Studies have shown that impaired bone quality and quantity in CKD are ascribed to decreased bone strength, which ultimately enhances the risk for bone fractures (Bacchetta et al. 2010; Malluche et al. 2011; Nickolas et al. 2011, 2013; West et al. 2015; Ott 2016). Secondary hyperparathyroidism, retention of uremic toxins, acidemia, hypogonadism, increased oxidative stress and inflammation, and malnutrition may partly contribute to impairment in bone quality and quantity (Nickolas et al. 2008; Kazama et al. 2013; Drüeke and Massy 2016). However, the pathogenesis behind the increased incidence of bone fractures among the CKD population still remains unclear. Identification of pathways that critically regulate bone pathology in CKD patients is required to prevent uremia‐related bone fractures.

The Wnt/*β*‐catenin signaling pathway plays a pivotal role in osteogenesis through the differentiation of mesenchymal stem cells into osteoblasts (Baron and Kneissel 2013). Binding of Wnt agonists to the receptor comprising frizzled and low‐density lipoprotein receptor‐related protein 5/6 phosphorylates glycogen synthase kinase (GSK)‐3*β* and results in prevention of ubiquitin‐mediated proteolysis of *β*‐catenin in the cytosol, leading to the translocation of *β*‐catenin into the nucleus and promotion of transcription of osteogenesis‐related genes. Because the activity of the Wnt/*β*‐catenin signaling pathway largely depends on the amount of *β*‐catenin, GSK‐3*β* plays a key role in regulating this pathway. We previously reported that GSK‐3*β* heterozygous knockout (GSK‐3*β* ^+/−^) mice displayed accelerated bone development and regeneration through the Wnt/*β*‐catenin signaling pathway (Arioka et al. 2013). GSK‐3 inhibitors have been reported to increase bone mass and strength and promote bone healing in diseased rodents (Clément‐Lacroix et al. 2005; Kulkarni et al. 2006; Marsell et al. 2012; Sisask et al. 2013; Arioka et al. 2014). Collectively, these results suggest that GSK‐3*β* is a promising target for the development of novel bone anabolic agents.

Recent study has shown that repression of the Wnt/*β*‐catenin signaling pathway is associated with bone abnormalities in CKD (Sabbagh et al. 2012). The study revealed that phosphorylated *β*‐catenin‐positive osteocytes increased in patients with CKD compared with participants without CKD. More recently, animal experiments have shown that inhibition of Wnt antagonists, which act upstream of GSK‐3*β*, increases bone volume in CKD models (Fang et al. 2014; Moe et al. 2015). These results suggest that activation of the Wnt/*β*‐catenin signaling pathway has the potential to increase bone volume in CKD. However, it remains unclear whether direct inhibition of GSK‐3*β* increases bone volume in CKD.

This study sought to determine whether inhibition of GSK‐3*β* increases bone volume in CKD. To this end, we used an adenine‐induced CKD model, GSK‐3*β* ^+/−^ mice, and lithium chloride (LiCl), a known inhibitor of GSK‐3 (Ryves and Harwood 2001) in our in vivo model and evaluated the effects of GSK‐3*β* repression on bone volume and parameters assessed using microcomputed tomography (micro‐CT). We used GSK‐3*β* ^+/−^ mice in the study as GSK‐3*β* homozygous knockout mice show the embryonic lethality phenotype because of hepatocyte apoptosis and ventricular septal defects (Hoeflich et al. 2000; Kerkela et al. 2008).

## Materials and Methods

### Ethical considerations and animal care

The study protocol was approved by the Committee of Ethics on Animal Experiments of Kyushu University (A26‐213‐0). Animal handling and procedures were carried out in compliance with the Guidelines for Animal Experiments, Kyushu University, and Law (No. 105) and Notification (No. 6) of the Japanese Government. Mice were housed in a climate‐controlled space on a 12‐h day/night cycle and allowed free access to food and water. All synthetic rodent diets were purchased from Oriental Yeast Co., Ltd (Tokyo, Japan).

### Generation of GSK‐3*β* ^**+/−**^ mice

GSK‐3*β* ^+/−^ mice were generated as described previously by Kimura et al. (2008). Briefly, using homologous recombination, we flanked the exon‐encoding catalytic domain of GSK‐3*β* with LoxP elements. Floxed GSK‐3*β* mice were crossed with mice expressing Cre recombinase under the control of the EIIa promoter, and their progeny were crossed with C57BL/6 mice. Heterozygous knockout of a GSK‐3*β* allele was confirmed by PCR using mouse genomic DNA, as described previously by Kimura et al. (2008).

### Experimental protocol

Eight‐week‐old male wild‐type C57BL/6 mice (*n* = 24) were randomly divided into three groups. Control mice (CNT, *n* = 8) were fed a control diet (calcium 1.0%, phosphate 1.2%; Oriental Yeast Co., Ltd, Tokyo, Japan) for 6 weeks. The remaining 16 mice were fed a diet containing 0.2% adenine (adenine‐diet) for 6 weeks. Eight mice were not treated (CKD, *n* = 8) and the other eight mice were treated with LiCl (Wako, Osaka, Japan) in drinking water (150 mg/L) for 6 weeks (CKD+LiCl, *n* = 8). For the fourth group, GSK‐3*β* ^+/−^ mice (*n* = 8) were fed an adenine‐diet for 6 weeks (CKD‐GSK‐3*β* ^+/−^) to determine the effect of GSK‐3*β* haploinsufficiency on bone volume and properties. The adenine‐induced CKD mouse model was used to recapitulate uremia‐related bone abnormalities because adenine‐induced uremic rat and mouse models show chronic progressive tubulointerstitial nephritis caused by accumulation of 2,8‐dihydroxyadenine crystals in renal tubules and interstitia (Yokozawa et al. 1986; Jia et al. 2013).

One day before euthanasia, mice were housed in metabolic cages for 24 h, and food and water intake and urine volume were recorded. Mice were euthanized on day 42, and their blood and femurs collected. Blood was clotted at room temperature for 1 h and the obtained serum was separated by centrifugation at 3000 × *g* and stored at −30°C until analysis. The left femur was immersed in 70% ethanol and stored at 4°C until analysis.

### Biochemical parameters

Serum concentrations of albumin, urea nitrogen, sodium, calcium, and phosphate were measured with an automated analyzer (Hitachi, Tokyo, Japan). Serum levels of intact parathyroid hormone (PTH) (Immutopics International, San Clemente, CA), osteocalcin (Biomedical Technologies, Stoughton, MA), and tartrate‐resistant acid phosphatase‐5b (TRACP‐5b) (Immunodiagnostic Systems, Gaithersburg, MD) were determined using commercially available mouse ELISA kits. The kits were used according to the manufacturer's instructions, and their qualities were within analytical levels.

### Determination of bone volume and parameters by micro‐CT

Morphological analysis of mouse femurs was performed using a micro‐CT system (Skyscan 1076 scanner; Skyscan, Konitich, Belgium), as described previously (Bouxsein et al. 2010). Briefly, scanning conditions were set to 48 kV, 201 μA, and 9 μm for one scan image. Three‐dimensional reconstruction of images was performed with InstaRecon/NRecon software (Skyscan). Two regions were quantitatively analyzed in mice: the cortical bone region from 2.0 to 2.5 mm above the growth plate at the distal metaphysis; and the trabecular bone region from 0.1 to 1.1 mm above the growth plate at the distal metaphysis. We calculated the following parameters: bone volume/total volume; trabecular number; trabecular thickness; trabecular separation; cortical thickness; cortical bone area; and total bone area. For each parameter, micro‐CT‐derived standard bone morphometry nomenclature, symbols, and units were used (Bouxsein et al. 2010).

### Statistical analysis

All statistical analyses were performed using JMP version 10.0 software (SAS Institute, Tokyo, Japan). Data are presented as mean ± SEM. Differences among groups were compared using one‐way analysis of variance, followed by Tukey–Kramer tests. For all tests, a two‐tailed *P‐*value <0.05 was considered statistically significant.

## Results

### Adenine‐induced CKD mice develop azotemia, hyperphosphatemia, and hyperparathyroidism

Body weight at week 6 was significantly lower in CKD, CKD‐GSK‐3*β* ^+/−^, and CKD+LiCl mice than in CNT mice. There were no significant differences in body weight among the three CKD groups. Food intake was significantly lower in CKD mice than in CNT mice, while there were no significant differences in food intake among the three CKD groups (Table 1). Serum levels of albumin were comparable among the four groups. Serum levels of urea nitrogen, phosphate, and intact PTH were significantly higher in CKD, CKD‐GSK‐3*β* ^+/−^, and CKD+LiCl mice than in CNT mice, and were comparable among the three CKD groups. The serum calcium concentration was significantly higher in CKD+LiCl mice than in CNT mice, and there were no significant differences among the three CKD groups (Table 2).

**Table 1:** Physical parameters of each group after 6 weeks of treatment

	CNT	CKD	CKD‐GSK‐3*β* ^+/−^	CKD+LiCl
Body weight, g	27.6 ± 1.0	18.5 ± 0.3*	18.0 ± 0.7*	20.3 ± 0.2*
Food intake, g/day	2.8 ± 0.1	2.2 ± 0.1*	2.2 ± 0.2	2.5 ± 0.2
Water intake, mL/day	5.1 ± 0.4	7.8 ± 0.3	7.6 ± 1.4	24.9 ± 2.1*, †, ‡
Urine volume, mL/day	1.3 ± 0.2	3.9 ± 0.5	3.2 ± 0.5	16.3 ± 1.8*, †, ‡

CNT, mice fed a control diet; CKD, mice fed a diet containing 0.2% adenine; CKD‐GSK‐3*β* ^+/−^, GSK‐3*β* ^+/−^ mice fed a diet containing 0.2% adenine; CKD+LiCl, mice fed a diet containing 0.2% adenine and given water containing LiCl (0.15 mg/mL). Data are mean ± SEM, and were compared using one‐way ANOVA followed by the Tukey–Kramer test. A two‐tailed *P*‐value <0.05 was considered statistically significant. *P < 0.05 versus CNT, ^†^ *P < *0.05 versus CKD, ^‡^ *P < *0.05 versus CKD‐GSK‐3*β* ^+/−^. CKD, chronic kidney disease; CNT, control; GSK‐3*β* ^+/−^, glycogen synthase kinase‐3 beta heterozygous knockout; LiCl, lithium chloride.

**Table 2:** Serum biochemistries of each group after 6 weeks of treatment

	CNT	CKD	CKD‐GSK‐3*β* ^+/−^	CKD+LiCl
Albumin, g/dL	3.2 ± 0.1	3.2 ± 0.1	3.0 ± 0.1	3.3 ± 0.1
Urea nitrogen, mg/dL	25.3 ± 1.5	59.1 ± 1.7*	63.9 ± 3.5*	54.3 ± 4.8*
Sodium, mEq/L	147.3 ± 1.3	148.5 ± 2.5	149.2 ± 2.5	148.6 ± 1.2
Calcium, mg/dL	8.5 ± 0.2	9.1 ± 0.2	8.9 ± 0.3	9.7 ± 0.2*
Phosphate, mg/dL	9.3 ± 0.5	15.6 ± 1.1*	16.2 ± 1.6*	15.3 ± 0.9*
Intact PTH, pg/mL	430 ± 134	2948 ± 403*	2942 ± 467*	2919 ± 381*
Osteocalcin, ng/mL	9.4 ± 2.9	101.2 ± 17.8*	140.8 ± 23.1*	76.9 ± 8.7*, ‡
TRACP‐5b, U/L	2.1 ± 0.5	9.0 ± 1.3*	9.8 ± 1.6*	8.2 ± 0.7*

CNT, mice fed a control diet; CKD, mice fed a diet containing 0.2% adenine; CKD‐GSK‐3*β* ^+/−^, GSK‐3*β* ^+/−^ mice fed a diet containing 0.2% adenine; CKD+LiCl, mice fed a diet containing 0.2% adenine and given water containing LiCl (0.15 mg/mL). Data are mean ± SEM, and were compared using one‐way ANOVA followed by the Tukey–Kramer test. A two‐tailed *P*‐value <0.05 was considered statistically significant. *P < 0.05 versus CNT, ^‡^ *P < *0.05 versus CKD‐GSK‐3*β* ^+/−^. CKD, chronic kidney disease; CNT, control; GSK‐3*β* ^+/−^, glycogen synthase kinase‐3 beta heterozygous knockout; LiCl, lithium chloride; PTH, parathyroid hormone; TRACP‐5b, tartrate‐resistant acid phosphatase‐5b.

### Bone turnover markers increase in adenine‐induced CKD mice

Serum levels of osteocalcin and TRACP‐5b were significantly higher in CKD, CKD‐GSK‐3*β* ^+/−^, and CKD+LiCl mice than in CNT mice. The serum concentration of osteocalcin was significantly lower in CKD+LiCl mice than in CKD‐GSK‐3*β* ^+/−^ mice. The serum levels of TRACP‐5b was comparable among the three CKD groups (Table 2).

### LiCl treatment induces polyuria and polydipsia

As shown in Table 1, water intake and urine volume at week 6 were significantly higher in CKD+LiCl mice than in CNT, CKD, and CKD‐GSK‐3*β* ^+/−^ mice. Serum levels of sodium were comparable among the four groups.

### Uremia affects cortical bone volume

**Figure 1:**
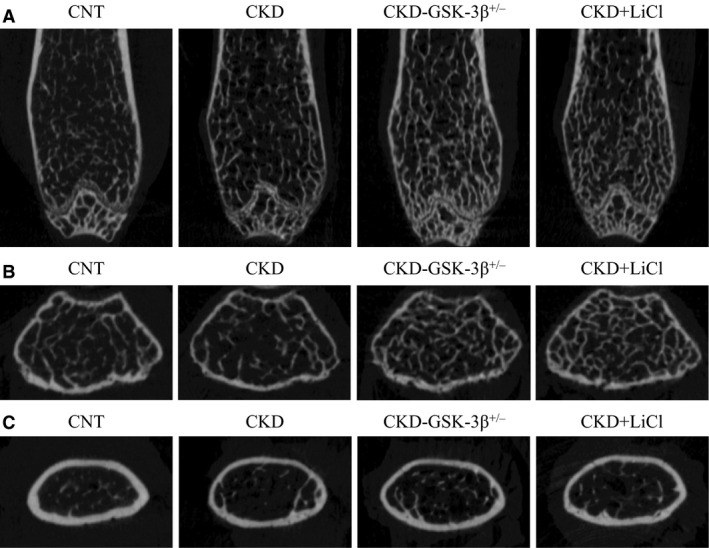
Representative microcomputed tomography images of femurs. (A) Longitudinal images of femurs. (B) Cross‐sectional images of femurs in trabecular bone area. (C) Cross‐sectional images of femurs in cortical bone area. CKD, chronic kidney disease; CNT, control; GSK‐3*β* ^+/−^, glycogen synthase kinase‐3 beta heterozygous knockout; LiCl, lithium chloride.

**Figure 2:**
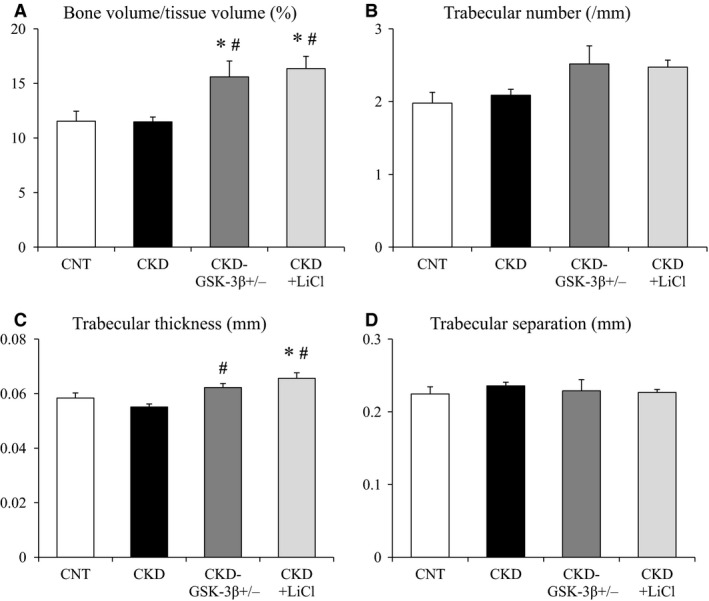
Quantitative morphological parameters for trabecular bone. (A) Bone volume/tissue volume (%), (B) Trabecular number (/mm), (C) Trabecular thickness (mm), and (D) Trabecular separation (mm). Data are mean ± SEM, and were compared using one‐way ANOVA followed by the Tukey–Kramer test. A two‐tailed *P*‐value <0.05 was considered statistically significant. *P < 0.05 versus CNT, ^#^ *P < *0.05 versus CKD. CKD, chronic kidney disease; CNT, control; GSK‐3*β* ^+/−^, glycogen synthase kinase‐3 beta heterozygous knockout; LiCl, lithium chloride.

**Figure 3:**
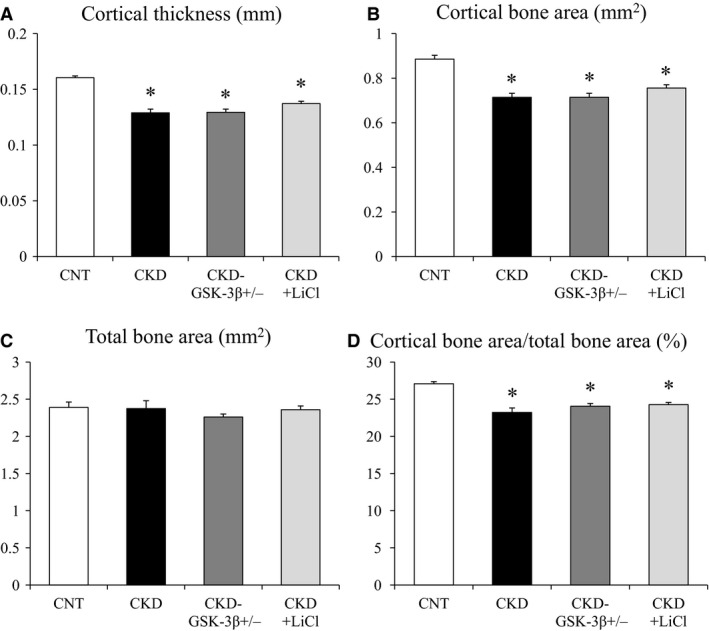
Quantitative morphological parameters for cortical bone. Cortical thickness (mm), (B) Cortical bone area (mm^2^), (C) Total bone area (mm^2^), and (D) Cortical bone area/total bone area (%). Data are mean ± SEM, and were compared using one‐way ANOVA followed by the Tukey–Kramer test. A two‐tailed *P*‐value <0.05 was considered statistically significant. *P < 0.05 versus CNT. CKD, chronic kidney disease; CNT, control; GSK‐3*β* ^+/−^, glycogen synthase kinase‐3 beta heterozygous knockout; LiCl, lithium chloride.

Representative coronal and cross‐sectional images of femurs from each group are shown in Figure 1. Quantitative morphological parameters for trabecular bone volume are shown in Figure 2. Bone volume/tissue volume, trabecular number, and trabecular thickness were not decreased in CKD mice compared with CNT mice (Fig. 2A–C). There were no significant differences in trabecular separation between CNT and CKD mice (Fig. 2D). Figure 3 shows quantitative morphological parameters for cortical bone volume. Cortical thickness, cortical bone area, and cortical bone area/total bone area were significantly reduced in CKD mice compared with CNT mice (Fig. 3A, B, and D). There were no significant differences in total bone area between CNT and CKD mice (Fig. 3C).

### GSK‐3*β* inhibition increases trabecular bone volume but does not affect cortical bone volume in CKD mice

Bone volume/tissue volume significantly increased in CKD‐GSK‐3*β* ^+/−^ and CKD+LiCl mice compared with CNT and CKD mice (Fig. 2A). Trabecular thickness was higher in CKD‐GSK‐3*β* ^+/−^ and CKD+LiCl mice than in CKD mice. In CKD+LiCl mice, in particular, trabecular thickness was elevated, even compared with CNT mice (Fig. 2C). Trabecular number tended to be high in CKD‐GSK‐3*β* ^+/−^ and CKD+LiCl mice, but there were no significant differences (Fig. 2B). Trabecular separation was comparable among the four groups (Fig. 2D). Cortical thickness, cortical bone area, and cortical bone area/total bone area were not significantly rescued in CKD‐GSK‐3*β* ^+/−^ and CKD+LiCl mice compared with CKD mice (Fig. 3A, B, and D). Total bone area was comparable among the four groups (Fig. 3C).

## Discussion

This study is the first to show the impact of GSK‐3*β* inhibition on bone volume in CKD mice. By using GSK‐3*β* ^+/−^ mice and LiCl, we revealed that inhibition of GSK‐3*β* increased trabecular bone volume in adenine‐induced uremic mice without affecting kidney function and mineral parameters, but did not improve the decrease in cortical bone volume. These findings suggest that GSK‐3*β* inhibitor serves as a bone anabolic agent that increases trabecular bone volume and potentially reduces the risk for bone fractures even in CKD.

In this study, GSK‐3*β* inhibition increased trabecular bone volume. Although cortical bone mass is a major determinant of bone strength and has been shown to be associated with bone fracture risk in patients on hemodialysis (Jamal et al. 2006), recent clinical studies have revealed that not only cortical bone volume but also trabecular bone volume is associated with the risk for fractures (Nickolas et al. 2010; Cejka et al. 2011; Bucur et al. 2015). Furthermore, bone volume/tissue volume has been shown to reflect trabecular bone connectivity, a component of bone quality (Kazama et al. 2010). Therefore, our results suggest that an increase in trabecular bone volume by GSK‐3*β* inhibition potentially reduces the risk for bone fractures in CKD. Notably, in our adenine‐induced CKD model, trabecular bone volume was not decreased in CKD group compared with CNT group, indicating that the increase in trabecular bone volume by GSK‐3*β* inhibition might be achieved independently of CKD‐related bone abnormalities. In this regard, we can only conclude that inhibition of GSK‐3*β* increases trabecular bone volume even under the CKD condition such as hyperphosphatemia and hyperparathyroidism. However, given that few bone anabolic agents are available especially in CKD, our data are promising and should be confirmed by other CKD models with decreased trabecular bone volume.

GSK‐3*β* ^+/−^ mice with normal kidney function showed increases in trabecular and cortical bone volume in a previous study (Arioka et al. 2013). In this study, however, inhibition of GSK‐3*β* increased trabecular bone volume, but did not rescue the decrease in cortical bone volume in adenine‐induced CKD mice. Similar findings were observed in CKD rats treated with anti‐sclerostin antibody, a known activator of the Wnt/*β*‐catenin signaling pathway (Moe et al. 2015). In that study, the researchers found that treatment with anti‐sclerostin antibody increased trabecular bone volume in CKD rats with low PTH but had no effect on cortical bone volume in CKD rats with low and high serum PTH levels. These results indicate that the magnitude of the serum PTH level influences the effects of the Wnt/*β*‐catenin signaling pathway on bone volume in CKD. It has also been reported that continuous elevation of PTH results in a reduction of bone volume (Frolik et al. 2003). In addition, phosphate and uremic toxin have been shown to suppress osteoblastic differentiation and induce osteoblast dysfunction (Tanaka et al. 2013; Okamoto et al. 2014). Taken together, the catabolic effect induced by sustaining high levels of PTH and osteoblast dysfunction by uremia might have exceeded the anabolic effect of GSK‐3*β* inhibition on cortical bone volume in our CKD mice. Further studies are necessary to determine whether GSK‐3*β* inhibition increases cortical bone volume in CKD model with controlled levels of PTH.

Differences in the effects of CKD on cortical and trabecular bone are an intriguing subject. One clinical study reported that CKD patients showed decreased cortical bone volume, which negatively correlated with the serum PTH level (Nickolas et al. 2013). Another study reported that the trabecular microarchitecture was impaired and trabecular bone volume decreased in CKD patients without severe hyperparathyroidism (Bacchetta et al. 2010). Notably, differential effects of hyperparathyroidism on trabecular and cortical bone were observed in a rat model of CKD (Miller et al. 1998). In that study, trabecular bone volume decreased in CKD rats with mild‐to‐moderate hyperparathyroidism, while it increased in CKD rats with severe hyperparathyroidism. Cortical porosity was positively associated with the serum PTH level. These results indicate that differences between changes in trabecular bone volume and cortical bone volume may be partly explained by the degree of hyperparathyroidism and bone turnover in CKD. As adenine‐induced CKD mice developed moderate‐to‐severe hyperparathyroidism, it is plausible that cortical bone volume decreased and trabecular bone volume did not change in our CKD mice. However, the precise mechanisms of changes in bone volume in CKD still remain unknown.

Serum bone metabolism markers are often used to estimate bone metabolism because bone volume is mainly determined by the balance between bone formation and resorption (Chu et al. 2003; Shidara et al. 2008). In this study, we measured serum levels of osteocalcin, a marker of bone formation, and TRACP‐5b, a marker of bone resorption. Both of them were increased in the three CKD groups compared with control group, showing high bone turnover induced by severe hyperparathyroidism as evidenced by high serum PTH levels. These changes in bone turnover markers also indicated that combined measurement of these two different bone metabolism markers could not explain the differential effect of GSK‐3*β* inhibition on cortical and trabecular bone volume in our study. This is partly because serum levels of bone turnover makers reflect the total summation of the systemic bone metabolism. Further experiments such as bone histomorphometric analyses are required to determine the differential impact of GSK‐3*β* inhibition on cortical and trabecular bone metabolism in CKD.

In this study, there were no significant differences between GSK‐3*β* haploinsufficiency and LiCl administration in terms of effect on bone volume. GSK‐3 has two isoforms, GSK‐3*α* and GSK‐3*β* (Woodgett 1990). Although homology of the two isoforms is 98% in the catalytic domain, their function is not identical. GSK‐3*β* homozygous knockout mice exhibit the embryonic lethality phenotype (Hoeflich et al. 2000; Kerkela et al. 2008), whereas GSK‐3*α* homozygous knockout mice are viable. In the Wnt/*β*‐catenin signaling pathway, functional redundancy of the two isoforms has been reported (Doble et al. 2007), and an increase in bone volume was observed in both GSK‐3*α* homozygous knockout mice (Zhou et al. 2013) and GSK‐3*β* ^+/−^ mice (Arioka et al. 2013). Because lithium inhibits both GSK‐3*α* and GSK‐3*β*, GSK‐3*β* ^+/−^ mice are not always similar to mice treated with LiCl. Although isoform‐specific inhibition is a challenging task, further studies are necessary to determine the differential effect of each GSK‐3 isoform‐specific inhibition on bone volume in CKD.

GSK‐3 is associated with the pathophysiology of several diseases, such as nervous system disorders, deranged glucose metabolism, nephrogenic diabetes insipidus, acute kidney injury, inflammation, cardiac hypertrophy, abnormal bone formation, vascular calcification, and cancer (Rao et al. 2010; Wang et al. 2010; Bao et al. 2012; Howard et al. 2012; Takahashi‐Yanaga 2013; Nørregaard et al. 2015). In this study, mice treated with LiCl developed polyuria and polydipsia, while GSK‐3*β* ^+/−^ mice did not. The difference may be explained by the fact that the effect of lithium on the ability to concentrate urine is mediated by dual inhibition of GSK‐3*α* and GSK‐3*β* (Rao et al. 2010; Nørregaard et al. 2015). In GSK‐3*β* ^+/−^ mice, residual GSK‐3*β* and GSK‐3*α* might maintain the ability to concentrate urine. With regard to the effects on kidney function, inhibition of GSK‐3 had no positive or negative effects in the adenine‐induced model. Kidney damage induced by adenine might exceed the renal effects of GSK‐3 inhibition (Wang et al. 2010; Bao et al. 2012; Howard et al. 2012). These results suggest that inhibition of this kinase is potentially double‐edged and involves a risk for unfavorable effects, especially if used long‐term.

Previous studies have shown that lithium affects parathyroid gland, kidney, and other organs and has the potential to cause several adverse effects such as hypercalcemia, hyperparathyroidism, chronic renal interstitial fibrosis, renal impairment, and nephrogenic diabetes insipidus (Rao et al. 2005; McKnight et al. 2012; Walker et al. 2013; Shine et al. 2015). Although there were no significant differences in kidney function and serum levels of PTH between CKD and CKD+LiCl mice, we could not exclude the possibility that lithium affected kidney and parathyroid function, thereby indirectly affecting bone metabolism in our study. In this regard, in patients with CKD, treatment with LiCl may not be practical because of its renal toxicity (Walker et al. 2013; Shine et al. 2015), potential induction of secondary hyperparathyroidism, and the difficulties in its dose adjustment. Thus, therapeutic approaches that exert bone‐specific inhibitory effects on GSK‐3*β* are more ideal, especially in the CKD population.

This study has several limitations. First, the effects of GSK‐3*β* inhibition on bone strength were not evaluated. Second, although we previously found a decrease in the expression of GSK‐3*β* and an increase in the expression of *β*‐catenin in bone marrow cells from GSK‐3*β* ^+/−^ mice (Arioka et al. 2013), we did not assess the expression of GSK‐3*β* or *β*‐catenin in GSK‐3*β* ^+/−^ mice and CKD mice with LiCl treatment. Third, the effects of GSK‐3 isoform‐specific inhibition on uremic bone volume should be clarified in future studies. Because the GSK‐3*β* homozygous knockout shows the embryonic lethality phenotype, osteocyte or osteoblast‐specific GSK‐3*β* deletion will be necessary. Forth, only male mice were studied in this study. Given that there is a crosstalk between GSK‐3*β* signaling and estrogen signaling pathway and the prevalence of osteoporosis in women with CKD is high (Klawansky et al. 2003; Yin et al. 2015), future studies should be directed toward the impact of GSK‐3*β* inhibition on bone in female mice and more specifically, in ovariectomized mouse model. Lastly, we determined the effects of GSK‐3*β* inhibition on the trabecular bone volume in only one CKD model. Because the effects of CKD on trabecular bone volume were not consistent among different CKD models (Lau et al. 2013; Ferrari et al. 2014; Liu et al. 2014), further studies are needed to examine the impact of GSK‐3*β* inhibition on the trabecular bone volume in other CKD models such as 5/6th nephrectomy.

In conclusion, this study found that inhibition of GSK‐3*β* increased trabecular bone volume, but did not rescue the decrease in cortical bone volume in adenine‐induced uremic mice. These results suggest that an inhibitor of GSK‐3*β* may be a potential candidate as a bone anabolic agent in the CKD population. Further studies are needed to determine the beneficial effects of GSK‐3*β* blocking on bone quality, quantity, strength, and fracture risk in CKD patients.

## Conflict of Interest

None declared.
